# Supplementary material for: Basigin drives intracellular accumulation of l-lactate by harvesting protons and substrate anions
Source: PLoS One. 2021 Mar 26;16(3):e0249110. doi: 10.1371/journal.pone.0249110 (PMC7996999; doi:10.1371/journal.pone.0249110)
Supplement: S1 Fig — Highlighted are the N-terminal hemagglutinin epitope tag (pink) and the C-terminal His10 affinity tag (blue), the cysteine residues forming disulfide bridges in the Ig-like domains Ig-I (dark grey) and Ig-C2 (light grey), the glutamate in the transmembrane helix of basigin (red), and the charged residues located in the negative (red) and positive patch (blue) of the Ig-I domain. The N-terminal signal peptide present in BSG var2 is shaded white. The expression constructs of this study were generated by truncations at the sites of changed grey levels between the extracellular domains (BSGΔIg, BSG Ig-I, BSG Ig I/C2, BSG var2); or lacked the basigin fusion altogether (MCT1). All contained an N-terminal hemagglutinin epitope-tag. (PDF) [file pone.0249110.s001.pdf]

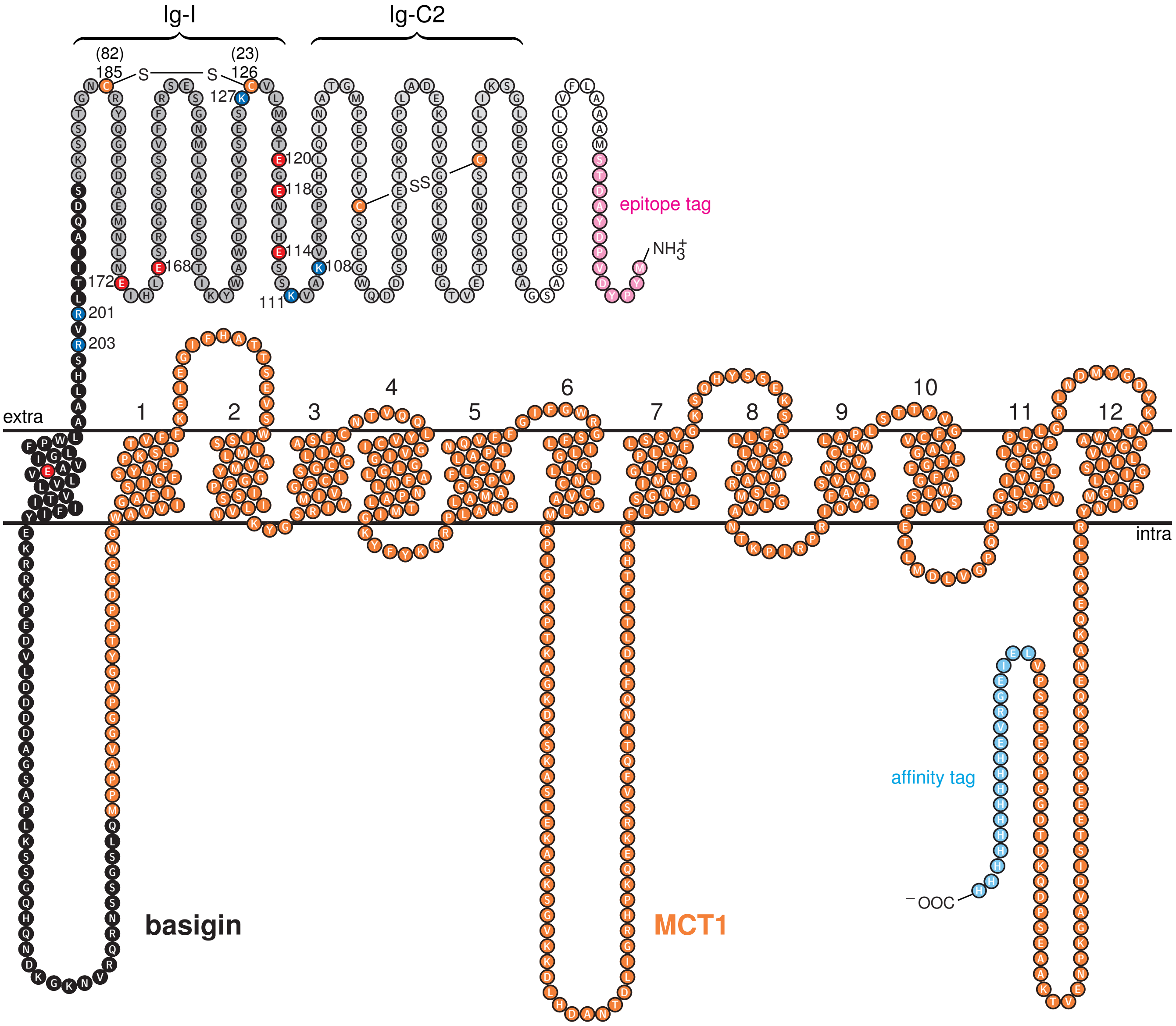


**Figure S1.** Topology and protein sequence of the MCT1 fusion constructs with basigin variants. Highlighted are the N-terminal hemagglutinin epitope tag (pink) and the C-terminal $His_{10}$ affinity tag (blue), the cysteine residues forming disulfide bridges in the Ig-like domains Ig-I (dark grey) and Ig-C2 (light grey), the glutamate in the transmembrane helix of basigin (red), and the charged residues located in the negative (red) and positive patch (blue) of the Ig-I domain. The N-terminal signal peptide present in BSG var2 is shaded white. The expression constructs of this study were generated by truncations at the sites of changed grey levels between the extracellular domains (BSGΔIg, BSG Ig-I, BSG Ig-I/C2, BSG var2); or lacked the basigin fusion altogether (MCT1). All contained an N-terminal hemagglutinin epitope-tag.
