# Supplementary material for: Basigin drives intracellular accumulation of l-lactate by harvesting protons and substrate anions
Source: PLoS One. 2021 Mar 26;16(3):e0249110. doi: 10.1371/journal.pone.0249110 (PMC7996999; doi:10.1371/journal.pone.0249110)
Supplement: S2 Fig — Jen1Δ ady2Δ yeast cultures with and without expression of MCT1 were switched from rich glucose conditions to liquid media containing 1% sodium lactate as the sole carbon source and growth was monitored by changes in the optical density (OD600). (PDF) [file pone.0249110.s002.pdf]

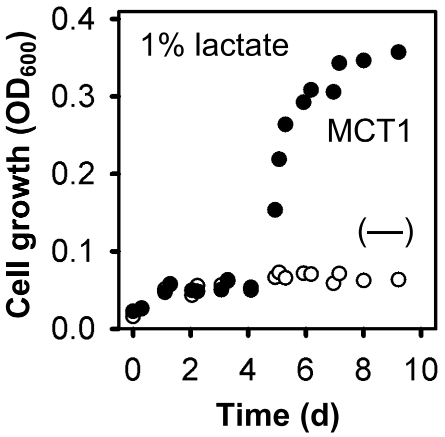

Figure S2. Delay of yeast growth during metabolic adaptation. *Jen1*Δ *ady2*Δ yeast cultures with and without expression of MCT1 were switched from rich glucose conditions to liquid media containing 1 % sodium lactate as the sole carbon source and growth was monitored by changes in the optical density (OD_{600}).
