# Supplementary material for: Basigin drives intracellular accumulation of l-lactate by harvesting protons and substrate anions
Source: PLoS One. 2021 Mar 26;16(3):e0249110. doi: 10.1371/journal.pone.0249110 (PMC7996999; doi:10.1371/journal.pone.0249110)
Supplement: S3 Fig — Shown are curves for BSG Ig-I/C2 E(27)R (▽) and BSG var2 E(27)R (△). The data were normalized to 1 mg of cells and the background of non-expressing cells was subtracted. Error bars indicate ± S.E.M. from three biological replicates. (PDF) [file pone.0249110.s003.pdf]

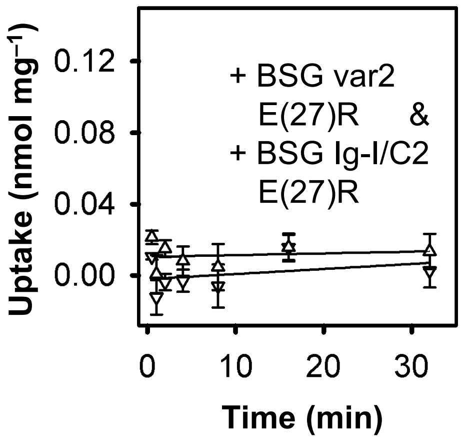

Figure S3. Uptake of ^{14}C-labeled L-lactate into *jen1*Δ *ady2*Δ yeast over time at pH 6.8 and a 1 mM inward gradient. Shown are curves for BSG Ig-I/C2 E(27)R (▽) and BSG var2 E(27)R (△). The data were normalized to 1 mg of cells and the background of non-expressing cells was subtracted. Error bars indicate ± S.E.M. from three biological replicates.
