# Supplementary material for: Basigin drives intracellular accumulation of l-lactate by harvesting protons and substrate anions
Source: PLoS One. 2021 Mar 26;16(3):e0249110. doi: 10.1371/journal.pone.0249110 (PMC7996999; doi:10.1371/journal.pone.0249110)
Supplement: S5 Fig — Glu → Gln (Glu114,118,120,168,172), Lys/Arg → Ala (Lys108,111,127 plus Arg201,203) or as indicated. (PDF) [file pone.0249110.s005.pdf]

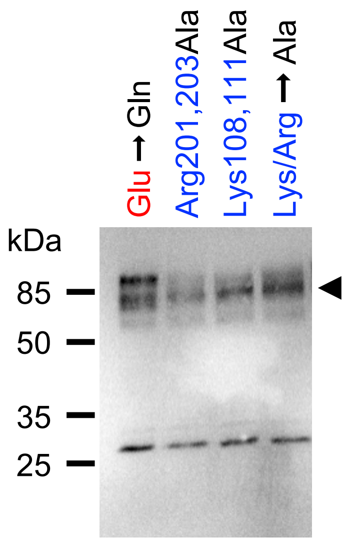

Figure S5. Western blot showing expression of MCT1 fusion constructs with BSG Ig-I carrying mutations of charged residues in the negative and positive patches. Glu → Gln (Glu114,118,120,168,172), Lys/Arg → Ala (Lys108,111,127 plus Arg201,203) or as indicated.
