# Supplementary material for: Basigin drives intracellular accumulation of l-lactate by harvesting protons and substrate anions
Source: PLoS One. 2021 Mar 26;16(3):e0249110. doi: 10.1371/journal.pone.0249110 (PMC7996999; doi:10.1371/journal.pone.0249110)
Supplement: S6 Fig — The structure data are from PDB# 6lz0. (PDF) [file pone.0249110.s006.pdf]

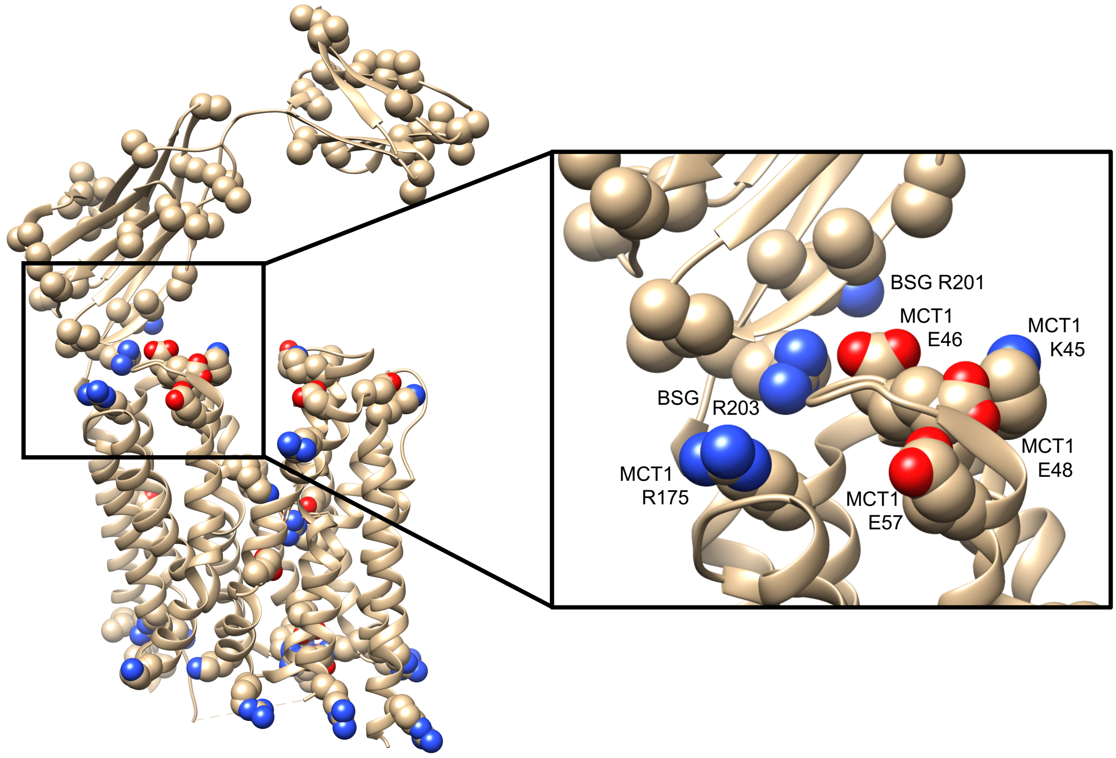

Figure S6. Charged residues at the interface between the basigin Ig-I domain (BSG; R201 and R203) and the extracellular side of MCT1 (E46, K45, E48, E57, R175). The structure data are from PDB# 6lz0.
