# Supplementary material for: Basigin drives intracellular accumulation of l-lactate by harvesting protons and substrate anions
Source: PLoS One. 2021 Mar 26;16(3):e0249110. doi: 10.1371/journal.pone.0249110 (PMC7996999; doi:10.1371/journal.pone.0249110)
Supplement: S1 Table — (PDF) [file pone.0249110.s007.pdf]

Table S1. Oligonucleotide list

Description	Sequence
MCT1 linker, sense	ctagtctgcagATGCCACCAGCAGTTGGTCCGGACCAGAAAGACA CAGATGGAGGGCCCAAGGAGGAGGAAAGTCCAGTCC
MCT1 linker, antisense	tcgaGGACTGGACTTTCCTCCTCCTTGGGCCCTCCATCTGTGTCT TTCTGGTCCGGACCAACTGCTGGTGGCATctgcaga
BSGΔIg, sense	ttactagtATGTCCGACCAGGCCATCATCACGC
BSGΔIg, antisense	ttctgcagggatccGGAAGAGTTCCTCTGGCGGACG
BSG Ig-I, sense	ttactagtatgCCCAGAGTGAAGGCTGTGAAGT
BSG Ig-I, antisense	ttctgcagggatccGGAAGAGTTCCTCTGGCGGACG
BSG Ig-I/C2, sense	ttactagtatggctgccGGCACAGTCTTCACTACCGTAG
BSG Ig-I/C2, antisense	ttctgcagggatccGGAAGAGTTCCTCTGGCGGACG
BSG var2, sense	ttactagtATGGCGGCTGCGCTGTTCGTGCTGCTGGGATTCGCGC TGCTGGGCACCCACGGAGCCTCCGGGGCTGCCgGCACAGTCTTCA CTACCGTAG
BSG var2, antisense	ttctgcagggatccGGAAGAGTTCCTCTGGCGGACG
E(27)R, sense	CTGGGCATCGTGGCTagaGTGCTGGTGCTG
E(27)R, antisense	AGCCACGATGCCCAGGAAGGGCCAGAGG
BSG Ig-I C23S, sense	ACGGCCATGCTGGTCTcCAAGTCAGAGTCCGTG
BSG Ig-I C23S, antisense	GACCAGCATGGCCGTCTCCCCCTCGTTGAT
BSG Ig-I C82S, sense	GGCCAGTACCGGTcCAACGGCACCAGCTCCAA
BSG Ig-I C82S, antisense	CCGGTACTGGCCGGGGTCGGCCTCCATGTT
BSG Ig-I Lys108,111Ala, sense	GCCCAGAGTGgcGGCTGTGgcGTCGTCAGAAC
BSG Ig-I Lys108,111Ala, antisense	CACTCTGGGCATACTAGTGTCCGCGTAGTCAGG
BSG Ig-I Arg201,203Ala, sense	CATCACGCTCgcCGTGgcCAGCCACCTGG
BSG Ig-I Arg201,203Ala, antisense	GAGCGTGATGATGGCCTGGTCGGAGCCCTTGG
