# Supplementary figures and images for: Basigin drives intracellular accumulation of l-lactate by harvesting protons and substrate anions
Source: PLoS One. 2021 Mar 26;16(3):e0249110. doi: 10.1371/journal.pone.0249110 (PMC7996999; doi:10.1371/journal.pone.0249110)

**Original uncropped and unadjusted images of Western blots**

**Fig. 1B**

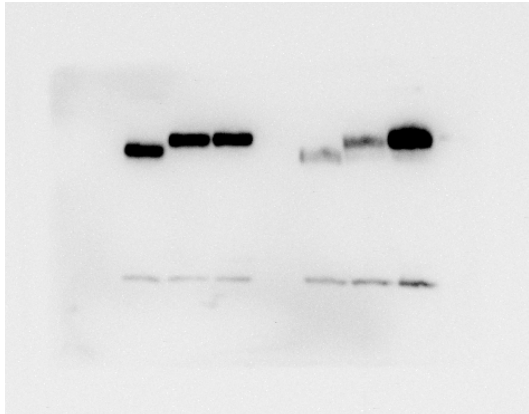

**Fig. 2A**

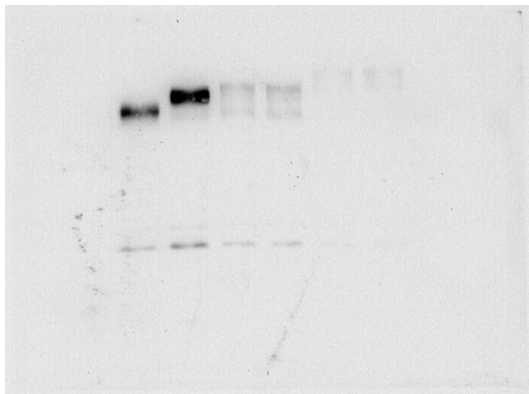

**Fig. 2F**

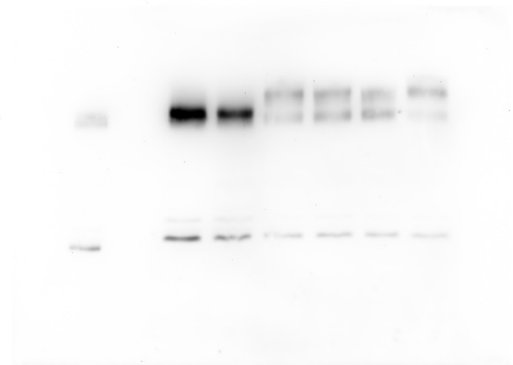

**Fig. S5**

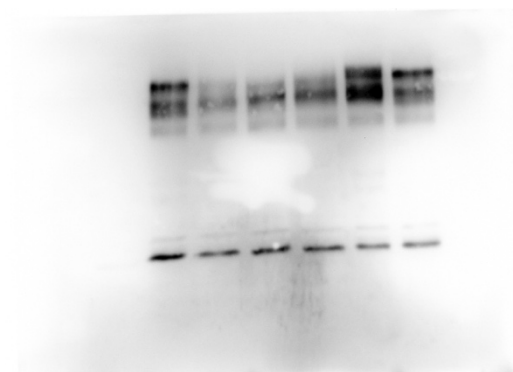

Supplement: S1 Raw images — (PDF) [file pone.0249110.s008.pdf]
