# Supplementary material for: Basigin drives intracellular accumulation of l-lactate by harvesting protons and substrate anions
Source: PLoS One. 2021 Mar 26;16(3):e0249110. doi: 10.1371/journal.pone.0249110 (PMC7996999; doi:10.1371/journal.pone.0249110)
Supplement: Attachment — Submitted filename: Review PONE-D-21-03063.pdf [file pone.0249110.s009.pdf]

Köpnick *et al.* report in their submitted manuscript PONE-D-21-03063 the role of the ancillary glycoprotein basigin on the intracellular accumulation of L-lactate.

Basigin associates with L-lactate-transporting members of the SLC16 family, which is also known as the monocarboxylate transporter (MCT) family, and mediates their trafficking to the plasma membrane. Basigin consists of one transmembrane helix, a long intracellular domain, as well as an extracellular domain, which is glycosylated and consists of different numbers of Ig-like domains (i.e. Ig-I, Ig-C2).

The authors established a *S. cerevisiae*-based assay, where they measure the uptake of radiolabeled L-lactate through concatemers consisting of MCT1 and versions of basigin (i.e., full-length and different truncations). Furthermore, the authors use the assay to monitor changes in the cytoplasmic pH upon exposure to L-lactate by using a pH-sensitive dye.

The authors show that the association of basigin with MCT1 leads to an intracellular accumulation of L-lactate, which is 4.5-fold above the substrate/proton concentrations provided by the external buffer.

The observed effect was assigned to the extracellular Ig-I domain of basigin by using basigin versions with truncated extracellular domains.

The authors identified surface patches of opposite (i.e., negative and positive) electrostatic potential on basigin, which they propose to act as proton and L-lactate binding sites, which locally increase substrate and co-substrate concentrations.

The authors conclude from their data that the Ig-I domain of basigin drives L-lactate uptake by locally increasing the proton and substrate concentrations at the extracellular side of MCT1.

Furthermore, the authors show that cell growth on media where L-lactate is the sole carbon source was strongly promoted in the presence of the Ig-I domain.

This manuscript is of interest for the readership of PLOS One and I recommend its publication after the following questions and concerns have been answered/addressed by the authors.

- The authors should provide line numbers in a revised version of the manuscript, which makes the review process much easier.

- The authors established a new transport assay using basigin-MCT1 concatemers. To my knowledge this has not been done before with any other member of the SLC16 family. Therefore, the authors should verify that the concatemers “behave” as expected by providing transport inhibition data using known and potent MCT1 inhibitors (e.g., AZD3965) and by providing kinetic data (i.e., Km-values and Vmax). Furthermore, it would be beneficial to provide transport data of the Arg313Ala, which should be non-functional.

- The authors must also cite the following publication: Grollman et al. Biochemistry 2000, 39, 9351, which describes the expression and functional

characterization of MCT3 in *S. cerevisiae*. This could be done on page 5: [25,26].

- From mammalian cells it is known that basigin is glycosylated. Did the authors also observe glycosylation of basigin when concatenated to MCT1 and/or when expressed alone? If multiple bands are detected on Western blots, sample treatment with de-glycosidases might be insightful.

- When comparing confocal microscopy images of control cells with images of yeast containing expressed MCT1 or concatemers (Fig. 1c and 2b), one can detect fluorescent circles within the yeast cells. Can the authors comment on the origin of these circular objects? Do they represent populations of MCT1 or concatemers, which are stuck in intracellular vesicles?

- In Fig. 5 the authors used the X-ray structure of the Ig-domains of basigin (i.e., BSG var2) to calculate the electrostatic surface potential and to detect charged clusters. However, in light of the now available cryo-EM structure of the MCT1-basigin heterodimer I strongly recommend computing the electrostatic surface potential of the heterodimer in order to check if the identified charged clusters have counter charges on the MCT1 transporter.

- Although the authors focus on the role of basigin, they should also mention that negatively-charged aspartate residues on MCTs have also been reported to play important roles in the transport activity and pH dependency. They might function as a first step of substrate and proton recognition: Yamaguchi *et al.* Biochem Biophys Res Commun. 2020 Sep 3;529(4):1061-1065

- On p. 4, the authors must provide a reference for their statement that basigin homologs are absent in yeast. How is MCT1 then trafficked to the plasma membrane?

- Did the authors compare the expression levels of GFP- and non-GFP-tagged MCT1? Do they share common transport characteristics? Why did the authors not use the GFP-tagged version also for transport measurements to allow direct comparison with confocal data?

- On p. 6 the authors claim that the BSGΔIg E27R-MCT1 fusion protein is well expressed and reached the plasma membrane. However, it does not mediate L-lactate transport. Do the authors conclude that E27 is directly important for L-lactate transport activity?

- For all presented constructs, the authors should provide membrane localization data in Table 1.

- In Table 1 the authors should provide Km and Vmax values for the presented constructs.

- In general, the authors should check their manuscript for consistency: total uptake or uptake capacity etc.

- On p. 6, the authors use the term “abscissa”. Do the authors mean the y- or x-axis?

- On p. 7, the authors mention that the Western blot indicates some protein fragmentation of the BSG Ig-I C23S and C82S constructs (Fig. 2F). This has also been observed for the normal Ig-I and Ig-I/C2 (Fig. 2A). The authors should perform Western blot analysis using antibodies against the basigin to check the identity of the observed bands.

- On p. 7, the authors claim that Cys-to-Ser mutations in the Ig-I domain render them misfolded. Is there any literature on the effect of disrupting the disulfide-bridges of the Ig-domains of basigin or related proteins? How can misfolded trafficking chaperones (i.e., basigin) mediate the proper trafficking of MCT1 to the plasma membrane? The authors must provide confocal microscopy data to show where the MCT1- BSG Ig-I C23S and C82S concatemers are located in the yeast. The authors should also measure L-lactate transport under reducing conditions, which also leads to breaking of the disulfide-bridges.

- On p. 8, the authors should give a reference for “8.6 x 10^6 cells per mg”.

- On p. 9, the authors mention “that the system was equilibrated”. However, this statement is only correct, if one assumes the highest measured intracellular L-lactate concentration of 0.44 mM. In the case of 0.18 mM, which was also measured, the system is not equilibrated.

- On p. 12; last line, the authors should modify their statement “by a factor of four” since the range of the factor is 0.3 – 3.5 according to the cited publication. Are the observed effects still virtually equal?

- On p. 13, I do not fully agree with the comparison of the here presented results with the effect of proteolytically modified basigin on intracellular lactate levels in lung cancer cells (ref. 16). In ref. 16 MCT4-mediated proton-coupled L-lactate efflux is studied whereas proton-coupled L-lactate uptake is studied in the here presented manuscript. MCT4-mediated proton-coupled L-lactate efflux requires an intracellular proton-binding site. However, in ref. 16 the mentioned protease functions on the extracellular side of basigin, which might not have anything to do with the intracellular proton binding.

- On p. 13, the authors should be more precise with the expression “L-lactate-releasing ….. cancers”. Do they mean cancer cells?

- On p. 14, there is a missing word in “The MCT1 ORF was cloned into using ….”

- Throughout the manuscript abbreviations – also of media etc. – should be introduced.

- On p. 15, replace “4.000 g” by “4,000 g”.

- On p. 15, please specify “twelve cycles”.

- On p. 15, please specify if the SDS-PAGE was done under reducing or non-reducing conditions.

- On p.16 and elsewhere, specify how the yeast cells were harvested (i.e., centrifugation speed etc.).

- On p. 17, was the uptake done under shaking and at which temperature?

- On p. 17, the authors mention “Michaelis-Menten kinetics”. However, no Km or Vmax values are reported in the manuscript. As far as I know, the specific activity is kept constant in Michaelis-Menten kinetics experiments (i.e., saturation curves), and not the amount of radioactivity as mentioned here: “final L-lactate concentrations of up to 10 mM were used each containing 0.1 uCi 14C-L-lactate”.

- On p. 17, the authors should mention for how long the scintillation counting was done.

- On p. 17, the authors should mention in the “Substrate transport assays” section how they converted the raw data (i.e., counts-per-minute) into “nmol”. Furthermore, it would be informative to mention the signal strength of a typical uptake experiment (i.e., counts-per-minute of untransformed yeast and MCT1-expressing yeast).

- On p. 17, the authors should provide a control experiment showing that the pH-sensitive dye remained inside the cell after loading.

- On p. 17, the authors should mention at which temperature the intracellular pH-measurements were done.

- On p. 18, the authors should mention the setting that were used for the Poisson-Boltzman electrostatics determination.

- On p. 18, the authors should specify for what they used the exponential fit (time-course?). What did they extract from the fit?

- Table 1: I recommend to provide amino acid numbers of all mutated residues.

- Fig 4A: The lines connecting the data points do not look like a fit while time-course experiments in other figures were always fitted using the described exponential model curve.
